# Social hierarchy modulates drug reinforcement and protein phosphorylation in the nucleus accumbens

**DOI:** 10.3389/fphar.2025.1537131

**Published:** 2025-04-11

**Authors:** Liang Xu, Ruiyi Zhou, Jiafeng Zhong, Yina Huang, Yingjie Zhu, Wei Xu

**Affiliations:** 1 West China School of Public Health and West China Fourth Hospital, Sichuan University, Chengdu, China; 2 Faculty of Life and Health Sciences, Shenzhen University of Advanced Technology, Shenzhen, China; 3 Shenzhen Key Laboratory of Drug Addiction, Shenzhen Neher Neural Plasticity Laboratory, The Brain Cognition and Brain Disease Institute, Shenzhen Institute of Advanced Technology, Chinese Academy of Sciences, Shenzhen, China; 4 University of Chinese Academy of Sciences, Beijing, China; 5 CAS Key Laboratory of Brain Connectome and Manipulation, The Brain Cognition and Brain Disease Institute, Shenzhen Institute of Advanced Technology, Chinese Academy of Sciences, Shenzhen, China

**Keywords:** social hierarchy, drug reinforcement, phosphoproteomics, drug addiction, HDAC4

## Abstract

**Introduction:**

Drug reinforcement, a form of behavioral plasticity in which behavioral changes happen in response to a reinforcing drug, would finally lead to drug addiction after chronical drug exposure. Drug reinforcement is affected by genetic and environmental factors. Social hierarchy has been reported to regulate drug reinforcement and drug-seeking behaviors, but the underlying molecular mechanism is almost unknown.

**Methods:**

We take advantage of the tube test to assess the social hierarchy between two co-housed rats. And then, we investigated the drug reinforcement between dominant and subordinate rats via conditioned place preference (CPP). Then we adopted 4-D label-free mass spectrometry to explore the complex phosphoproteome in the nucleus accumbens (NAc) between dominant and subordinate rats. Functional enrichment, protein-protein, motif analysis and kinase prediction interaction analysis were used to investigate the mechanism between substance use disorder and social hierarchy. Specifically, we identified histone deacetylase 4 (HDAC4) which has been previously shown to play critical roles in drug addiction as a key node protein by phosbind-SDS. Finally, we forcibly altered the social hierarchy of rats through behavioral training, follow by which we accessed the HDAC4 phosphorylation levels and drug reinforcement.

**Results:**

In this study, we found that methamphetamine exhibited stronger reinforcement in the subordinate rats. We identified 660 sites differing between dominant and subordinate rats via 4-D label-free mass spectrometry. Functional enrichment and protein-protein interaction analysis revealed that synaptic remodeling related pathways and substance use disorder related pathway are significantly characterized by social hierarchy. Motif analysis and kinase prediction showed that CaMKIIδ and its downstream proteins maybe the central hub. Phosbind-SDS revealed that higher HDAC4 phosphorylation levels in dominants. After the social hierarchy of rats were forcibly altered by behavioral training, the differences in HDAC4 phosphorylation levels induced by social hierarchy were eliminated, correspondingly the drug reinforcement is also reversed between the two group rats.

**Discussion:**

In conclusion, our research proves that protein phosphorylation in the NAc may be a vital link between social hierarchy and drug reinforcement.

## Introduction

Addiction is a chronic and relapsing disorder characterized by compulsive seeking and taking of a drug, even in the presence of adverse consequences (Koob and Volkow, 2016; Nestler and Landsman, 2001). However, drug use does not inevitably lead to addiction; only 15%–17% of individuals who use drugs progress to this condition (Swendsen and Le Moal, 2011). The transition from drug use to addiction is influenced by a complex interplay of factors, including drug pharmacology, social and environmental contexts, and genetic and epigenetic vulnerabilities (Bardo et al., 2013a; Volkow et al., 2019). Among these, social hierarchy—a ubiquitous phenomenon in both humans and animals that influences physiology and health (Sapolsky, 2005) and governs access to critical resources such as food and mates—has garnered significant attention (Milewski et al., 2022). Evidence suggests that individuals like monkey occupying lower social ranks, or subordinates, are more prone to drug abuse (Gould et al., 2017; Morgan et al., 2002; Nader et al., 2008), although the mechanisms underlying this increased vulnerability remain poorly understood.

The nucleus accumbens (NAc), a key hub in the regulation of addiction, motivation, and social hierarchy,is the site where all drugs with addictive potential increase dopamine (DA) levels, either directly or indirectly. This increase activates low-affinity dopamine D1 receptor medium spiny neurons (MSNs) (Castro and Bruchas, 2019; Choi et al., 2024; Volkow et al., 2019). Additionally, the NAc is well-known as an interface between motivational salience and behavioral output. Functional changes in the NAc caused by drug abuse continue to evolve during the abstinence stage, contributing to drug-seeking behavior and relapse due to emotional and motivational alterations (Schall et al., 2021). Neurons from the medial prefrontal cortex (mPFC) that project to the NAc (mPFC-NAc) encode social winning behavior (Choi et al., 2024). Recent studies have also revealed that the metabolic profile of the NAc is associated with social hierarchy (Larrieu et al., 2017).

Protein phosphorylation is a critical post-translational modification (PTM) that regulates diverse cellular processes, including signal transduction, transcription, apoptosis, and autophagy, and is implicated in various neuropsychological disorders (Bilbrough et al., 2022). It also plays a pivotal role in molecular networks underlying drug addiction (Lee and Messing, 2008). The phosphorylation of membrane receptors, such as μ-opioid peptide receptors (MOR) and AMPA receptors, modulates their activity and signal transduction efficiency, thereby influencing drug tolerance and addictive behaviors (Cadet et al., 2015; Duarte and Devi, 2020; Kibaly et al., 2016). For instance, the phosphorylation of GluA1-Ser831by CaMKII in the caudate and putamen (CPu) is essential for behavioral sensitization following nicotine exposure in rats (Kim et al., 2022).

Protein kinases and phosphatases are central to maintaining phosphorylation homeostasis. Notably, numerous studies have demonstrated that drugs of abuse can activate protein kinases, leading to alterations in gene transcription and protein synthesis (Bernstein et al., 2024). These molecular changes contribute to long-term modifications in synaptic function and neural circuitry, ultimately driving the development and persistence of addiction (Lee and Messing, 2008).

Recent studies highlight a significant interplay between social hierarchies and protein phosphorylation. For example, subordinate mice exhibit increased phosphorylation of the NMDA receptor subunit GluN2A and extracellular signal-regulated kinase 1/2 (ERK1/2) in the brain (Yoshida et al., 2022). Similarly, subordinate rainbow trout display heightened activity of liver AMP-activated protein kinase (AMPK), evidenced by an elevated phosphorylated AMPK ratio (Gilmour et al., 2017). Moreover, subordinate rats subjected to repeated defeat stress show increased ERK phosphorylation in the ventral tegmental area (VTA), a key brain region implicated in reinforcement and reward processes (Yap et al., 2015). These findings suggest that protein phosphorylation is influenced by social rank and may intersect with behaviors linked to drug addiction (Yap et al., 2015). What’s more, protein phosphorylation profiles have been identified play a role in drug memory extinction and reconsolidation in the NAc (Torregrossa et al., 2019). Research has shown that protein phosphorylation profiles related to cocaine memory extinction and reconsolidation change in the NAc, but there is no evidence indicating changes in protein phosphorylation profiles in the VTA or prefrontal cortex in the context of drug addiction (Choi et al., 2024). However, a comprehensive understanding of the phosphorylation landscape within the context of social hierarchies remains largely uncharted.

In this study, we utilized 4D label-free LC-MS to investigate the proteomic and phosphoproteomic profiles in the NAc of dominant and subordinate animals. While no significant differences were observed in overall protein expression between dominant and subordinate rats, the phosphoproteomic profiles revealed substantial variation. Furthermore, methamphetamine (Meth) demonstrated a more pronounced reinforcing effect in subordinate rats compared to dominant rats at the same dosage. Notably, differentially phosphorylated proteins were enriched in pathways associated with synaptic transmission and neural plasticity, both of which are critical in the development of drug addiction. To validate the phosphoproteomic findings, Phosbind SDS-PAGE analysis was performed. Additionally, behavioral interventions aimed at reversing social hierarchy were shown to modulate both drug reinforcement and protein phosphorylation in rats.

## Materials and methods

### Animals and drugs

Male Sprague-Dawley rats, aged 5–6 weeks, were obtained from Charles River (Beijing, China) for this study. The animals were housed under controlled conditions at a temperature of 22 °C–25 °C with a 12-hour light–dark cycle. All experimental procedures and animal care protocols were approved by the Animal Care and Use Committee of the Shenzhen Institute of Advanced Technology (SIAT), Chinese Academy of Sciences (CAS). The methamphetamine (>99% purity, PE- 0846) used in this study was purchased from Shanghai Yuansi Standard Science and Technology Co., Ltd.

### Tube test

The tube test, adapted from the previous deviously described method (Fan et al., 2023), was used to assess social hierarchy in rats. Starting at 5–6 weeks of age, animals were pair-housed with bodyweight-matched conspecifics for 2 weeks and handled daily for 7 days prior to testing. An 80-cm acrylic tube with a 60 mm internal diameter was used. Rats were trained to traverse the tube alone, completing 10 trials per day over 2 days, with counterbalanced starting positions. For formal testing, two mice were introduced at both ends and released as they meet in the middle of the tube. The rat that retreated or was pushed out was classified as the “subordinate” or “loser”, while the other was the “dominant” or “winner”. Each pair underwent 6 tests per day for 6 days, with social rank determined by the number of wins. Only pairs with consistent results across at least three consecutive days were included in subsequent experiments. Pairs with inconsistent outcomes were excluded.

### Forced win/loss

After establishing stable social ranks among paired rodents, rats were subjected to a forced win/loss protocol. This involved blocking the side of subordinate rats in the tube, leaving them with no choice but to push forward against their dominant counterpart. In cases where pairs remained locked in a standoff for more than 10 min, gentle force was applied behind the subordinate to encourage them to push the dominant rat out of the tube. This forced win/loss regimen consisted of 4 trials daily for a minimum of 2 weeks, continuing until the original subordinate rats could achieve wins without intervention. Rats that failed to alter their social rank after the forced win/loss sessions were excluded from subsequent formal experiments.

### Conditioned place preference (CPP)

The place preference apparatus comprised two distinct conditioning environments. Each conditioning environment measured 30 (L) × 30 (W) × 30 (H) cm. One environment had a floor consisting of smooth acrylic sheet and walls with alternating 2 cm wide black and white stripes. For another, the floor was rough acrylic sheet, and walls was black and white squares. Rats were handled and weighed the day before the start of an experiment. The activity of each rat was recorded and analyzed by AnyMaze software. On day 1, all rats were placed individually in the conditioned place preference apparatus and allowed to freely explore the entire apparatus for 30 min for acclimatization. On day 2 and 3, Animals were allowed to explore both sides of a custom-designed conditioned place preference (CPP) apparatus for 15 min to establish baseline preferences. Any rat that spent <20% or >80% of the entire time in either environment was removed from the study. During CPP training, animals received an intraperitoneal (*i.p.*) injection of methamphetamine (METH, 0.5 mg/kg) and were confined to their non-preferred chamber for 45 min before being returned to their home cage. The concentration of METH was 0.5 mg/mL. At least 8 h later, the same animals received an *i. p.* Injection of saline and were confined to their preferred chamber for 45 min. This alternating regimen of saline and drug injections was repeated over eight consecutive days. Twenty-four hours after the final training session, animals were re-exposed to the CPP apparatus and allowed to explore both sides freely for 15 min. The CPP score for each animal was determined by subtracting the baseline time spent in the drug-paired chamber from the test time.

### Mass spectrometry sample preparation

Sample preparation was similar between proteomics and phosphoproteomics. In brief, brain tissues were homogenized in liquid nitrogen and transferred to a 5 mL centrifuge tube. Four volumes of lysis buffer (8 M urea, 1% protease inhibitor cocktail) were added to the homogenized tissue, followed by sonication on ice using a high-intensity ultrasonic processor (Scientz). For post-translational modification (PTM) experiments, additional inhibitors were included in the lysis buffer. The homogenate was centrifuged at 12,000 g for 10 min at 4°C, and the resulting supernatant was collected. Protein concentration was measured using the BCA kit according to the manufacturer’s protocol.

For protein digestion, the solution was reduced with 5 mM dithiothreitol at 56°C for 30 min and alkylated with 11 mM iodoacetamide at room temperature for 15 min in the dark. The sample was then diluted with 100 mM triethylammonium bicarbonate (TEAB) to reduce the urea concentration less than 2 M. Trypsin was added at a 1:50 trypsin-to-protein mass ratio for overnight digestion, followed by a second digestion at a 1:100 ratio for 4 h. The resulting peptides were desalted using a C18 solid-phase extraction (SPE) column.

For biomaterial-based post-translational modification (PTM) enrichment in phosphoproteomics, peptide mixtures were incubated with an IMAC microsphere suspension in a loading buffer (50% acetonitrile and 0.5% acetic acid) under vibration. Non-specifically adsorbed peptides were removed through sequential washes with 50% acetonitrile/0.5% acetic acid and 30% acetonitrile/0.1% trifluoroacetic acid. Enriched phosphopeptides were eluted using an elution buffer containing 10% NH_4_OH, under vibration. The supernatant containing phosphopeptides was collected and lyophilized for subsequent LC-MS/MS analysis.

### LC-MS/MS analysis

All samples were analyzed by 4D mass spectrometer in proteomics and phosphoproteomics. Tryptic peptides were dissolved in solvent A (0.1% formic acid and 2% acetonitrile in water) and loaded onto a custom-made reversed-phase analytical column (25 cm length, 75/100 μm i. d.). Peptide separation was performed using a nanoElute UHPLC system (Bruker Daltonics) with the following gradient of solvent B (0.1% formic acid in acetonitrile): 6%–24% over 70 min, 24%–35% over 14 min, then increased to 80% over 3 min, and held at 80% for an additional 3 min. The flow rate was maintained at 450 nL/min throughout the run.

Peptides were analyzed using the timsTOF Pro mass spectrometer (Bruker Daltonics) under the following conditions: electrospray voltage set to 1.60 kV, and a scan range from 100 to 1700 m/z for precursor and fragment ions analyzed by the TOF detector. The instrument operated in parallel accumulation–serial fragmentation (PASEF) mode, performing 10 PASEF-MS/MS scans per cycle. Dynamic exclusion was set to 30 s.

MS/MS data were processed using the MaxQuant search engine (v.1.6.15.0). Tandem mass spectra were searched against the human SwissProt database (20,422 entries) concatenated with reverse decoy database. Trypsin/P was specified as the cleavage enzyme, allowing up to two missed cleavages. Mass tolerances for precursor and fragment ions were set to 20 ppm for the first search, 5 ppm for the main search, and the mass tolerance for fragment ions was set as 0.02 Da. Carbamidomethylation on cysteine was specified as a fixed modification, while acetylation of the protein N-terminus and oxidation of methionine were set as variable modifications. The false discovery rate (FDR) was controlled at < 1%.

### Functional enrichment

We used Kyoto Encyclopedia of Genes and Genomes (KEGG) database for KEGG pathway enrichment analysis. Fisher’s exact test was used to analyze the significance of KEGG pathway enrichment of differentially expressed proteins (using the identified protein as the background), and *P* value <0.05 were considered significant.

Gene Ontology (GO) annotations were categorized into Biological Process, Cellular Component, and Molecular Function. GO enrichment analysis of differentially expressed proteins was conducted using Fisher’s exact test, with a significance threshold of *P* < 0.05.

Protein domain enrichment analysis was performed using the Pfam database, with statistical significance determined by Fisher’s exact test (P < 0.05).

### Enrichment-based clustering

For further hierarchical clustering based on differentially expressed protein functional classification (such as: GO, Domain, Pathway, Complex). We first collated all the categories obtained after enrichment along with their *P* values, and then filtered for those categories which were at least enriched in one of the clusters with *P* value <0.05. This filtered *P* value matrix was transformed by the function x = −log10 (*P* value). These *P* values were then clustered by one-way hierarchical clustering (Euclidean distance, average linkage clustering) in Genesis. Cluster membership was visualized by a heat map using the “heatmap” function from the “ggplot2” R-package.

### Protein-protein interaction network

Protein-protein interactions were retrieved from the STRING database (version 11.5), focusing on interactions with a confidence score ≥0.7. Interaction networks were visualized using the R package “networkD3.”

### Protein phosphorylation-state analysis

The motif characteristics of modification sites were analyzed using the MoMo tool, which is based on the motif-x algorithm. Peptide sequences containing six amino acids upstream and downstream of all identified phosphorylation sites were included in the analysis. A motif was considered significant if it encompassed more than 20 peptide sequences and had a p-value less than 0.000001.

### Protein extraction and immunoblotting

Brain tissues were processed according to the manufacturer’s protocol using the N-PER™ Neuronal Protein Extraction Reagent (Thermo Fisher Scientific, #87792). To preserve protein integrity, a protease inhibitor cocktail (Thermo Fisher Scientific, #78442) and phosphatase inhibitors (Roche, #4906837001) were included. Protein concentrations were quantified using the Pierce™ BCA Protein Assay Kit (Thermo Fisher Scientific, #23227).

Proteins (20–30 μg) were separated by 7.5% sodium dodecyl sulfate–polyacrylamide gel electrophoresis (SDS-PAGE; EpiZyme, Shanghai, China) according to their molecular weight and transferred to a polyvinylidene fluoride (PVDF) membrane (Millipore, ISEQ00010). After blocking with 5% nonfat milk in TBST for 1 h at room temperature, the membranes were incubated overnight at 4°C with primary antibodies, followed by incubation with HRP-conjugated secondary antibodies for 2 h at room temperature. Blots were visualized using ECL 2 (Millipore, #WBKLS0500) and analyzed with a ChemiDoc XRS + system (Bio-Rad, Unites States). The primary antibody used was HDAC4 polyclonal antibody (Invitrogen; dilution 1:500), and the secondary antibody was goat anti-rabbit IgG (H + L), HRP conjugate (Invitrogen, A16104; dilution 1:1,000). Immunoblotting experiments were performed in three times to ensure reproducibility.

### Phospho-tag SDS-PAGE

For phospho-tag SDS-PAGE, proteins (60 μg) were separated using 6% SDS-PAGE (Beyotime, Jiangsu, China) containing Phos-tag Acrylamide (APEXBIO, Unites States). Phosphate ions in the proteins were captured by Phosbind, allowing separation based on the number of phosphate groups. Following electrophoresis, the gel was washed three times with EDTA (dissolved in transfer buffer) and once with transfer buffer. Proteins were then transferred to a polyvinylidene fluoride (PVDF) membrane (Millipore, ISEQ00010). The subsequent steps were performed as in the standard immunoblotting procedure.

### Statistics

All data are presented as mean ± standard deviation (SD). Statistical analyses were performed using GraphPad Prism (version 8.0) and SPSS (version 23.0). The Shapiro–Wilk test was used to assess the normality of data distribution. Differences between groups were evaluated using a Student’s t-test or paired *t*-test, as appropriate. A *P*-value of <0.05 was considered statistically significant.

## Results

### Social hierarchy affects drug reinforcement in male rats

**FIGURE 1:**
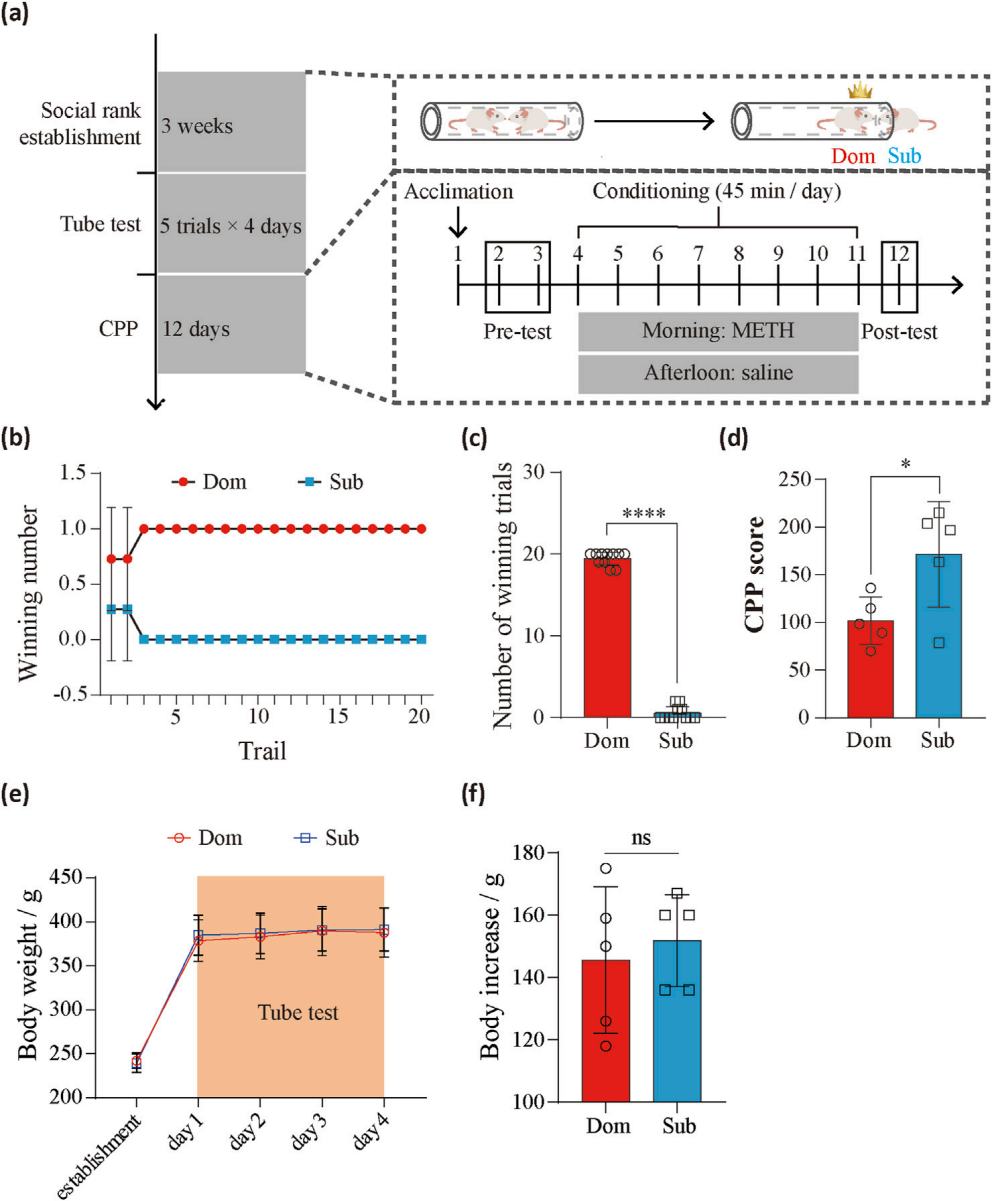
The effects of social competition on drug susceptibility. **(a)** Experimental timeline and schematics showing the tube test and CPP behavioral paradigms. **(b)** The average number of wins and losses for rats with different social status (*n* = 9 pairs) during the tube test. **(c)** Overall comparison of win and loss counts (*n* = 9 pairs, paired *t*-test, *t* (10) = 38.23, *P* < 0.0001). **(d)** Comparison of CPP scores between dominants (*n* = 5) and subordinates (*n* = 5) (Unpaired *t*-test, *t* (8) = 2.572, *P* = 0.033). **(e)** The change of body weight during tube test. **(f)** Comparison of body increase between dominants (*n* = 5) and subordinates (*n* = 5) (paired *t*-test, *t* (4) = 0.5369, *P* = 0.6198) after tube test. *P < 0.05. ****P < 0.0001. Data represent means ± SD. Dom, dominants. Sub, subordinates.

The tube test, a widely used model for assessing social hierarchy in rodents (Fan et al., 2019), was employed to evaluate the social hierarchy between cohabitating rats (Figure 1A). Four-week-old male rats were group-housed for 3 weeks to establish stable social ranks. A 4-day tube test protocol, consisting of five trials per day, was then conducted, leading to the identification of rats with consistent social ranks (Paired *t*-test, *t* (10) = 38.23, *P* < 0.0001) (Figures 1B, C). Rats were classified as “dominants” or “subordinates” based on their performance in the test. Following the tube test, the rats were divided into two groups. One group underwent conditioned place preference (CPP) testing to assess vulnerability to methamphetamine (METH), while the other group was subjected to proteome and phosphoproteome analyses. CPP was implemented to measure the reinforcing effects of METH. Interestingly, subordinate rats demonstrated significantly higher CPP scores compared to dominant rats (Unpaired *t*-test, *t* (8) = 2.572, *P* = 0.033) (Figure 1D). We recorded the body weight at the time of social hierarchy establishment and tube test. There were no significantly different in the body weight and body increase between the dominant and subordinate groups (paired *t*-test, *t* (4) = 0.5369, *P* = 0.6198) (Figures 1E, F). These findings suggest that social hierarchy may influence the reinforcing effects of METH in male rats.

### The phosphoproteomics of the NAc region in the dominants group showed significant difference compared to the subordinates group

**FIGURE 2:**
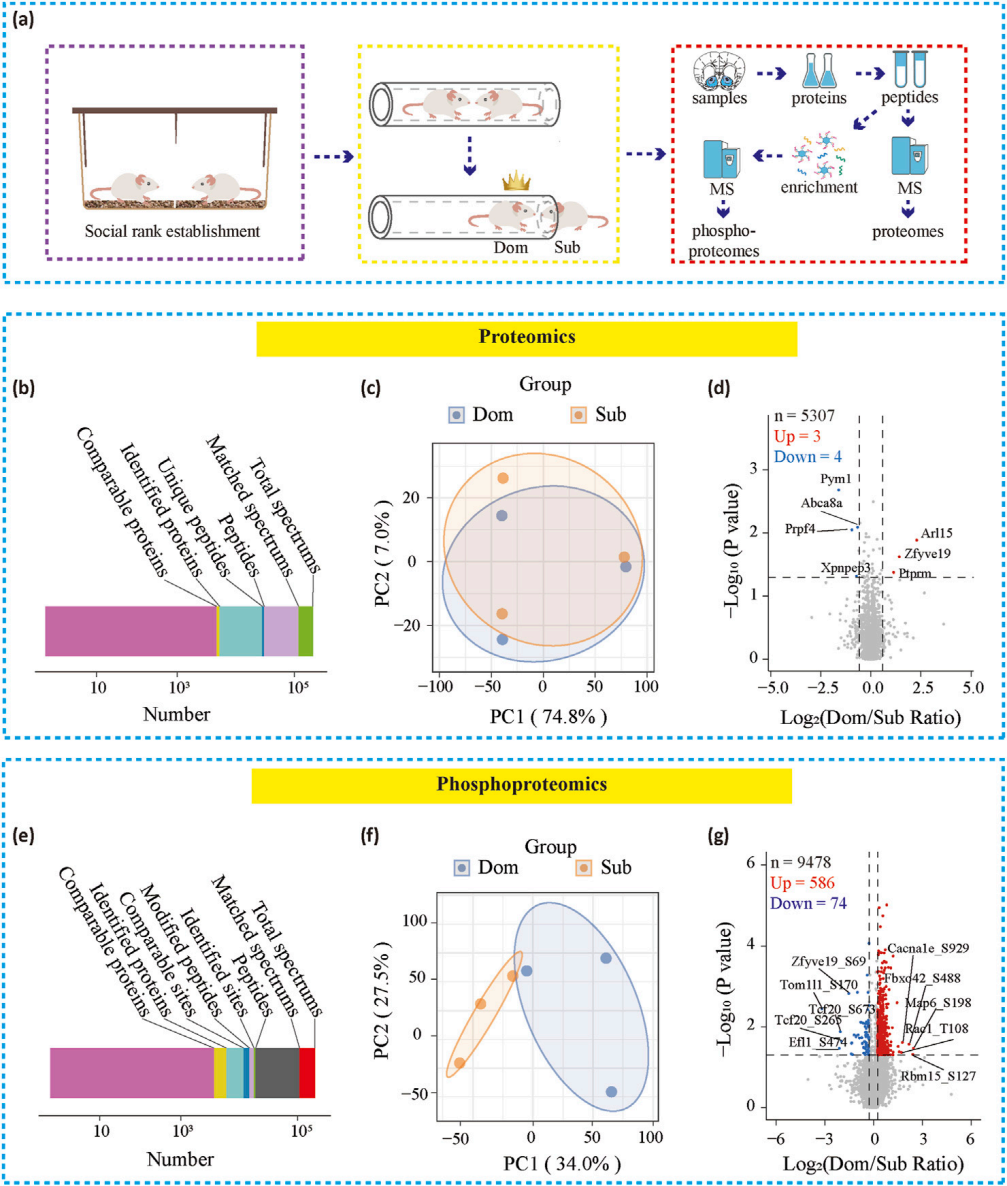
Proteomic and phosphoproteomics in paired rats. **(a)** Timeline showing proteomic and phosphoproteomics analysis **(b, c)** Overview of protein identification in proteomics or phosphoproteomics **(c, f)** PCA analysis for proteomics or phosphoproteomics **(d, g)** Volcano plots showing differential proteins or phosphorylated proteins of the NAc. Dom, dominants. Sub, subordinates.

To investigate the molecular mechanisms underlying social hierarchy and drug reinforcement, we performed proteomic and phosphoproteomic analyses of the nucleus accumbens (NAc), a critical brain region involved in drug addiction and social behavior (Koob and Volkow, 2016) (Figure 2A). We selected 9 pairs of rats with stable social hierarchies, categorized into dominant and subordinate groups. For each group, the NAc regions from 3 rats were pooled into one sample for subsequent analysis.

In the proteomic analysis, we identified a total of 52,620 unique peptides from 6,170 proteins, with 5,307 of these being comparable between the groups (Figure 2B). The phosphoproteomic analysis identified 15,535 phosphorylation sites across 4,201 proteins, of which 9,478 sites were comparable, including 2,327 comparable proteins (Figure 2E).

Principal component analysis (PCA) at the protein expression level failed to distinguish between the dominant and subordinate groups (Figure 2C). However, at the phosphoprotein expression level, PCA successfully differentiated the two groups (Figure 2F), indicating notable differences in phosphorylation modifications in the NAc between the groups. Correspondingly, the volcano plot of the proteomic data revealed that among the 5,307 proteins detected, only 7 showed significant differences between the dominant and subordinate groups (Figure 2D). In contrast, the phosphoproteomic analysis identified 660 phosphorylation sites that differed between the two groups: the dominant group exhibited 586 upregulated and 74 downregulated phosphorylation sites compared to the subordinate group (Figure 2G). These results suggest that phosphorylation modifications in the NAc play a critical role in social hierarchy and drug reinforcement.

### Functional classification analyses and protein-protein interaction (PPI) reveals synaptic remolding in social hierarchy

**FIGURE 3:**
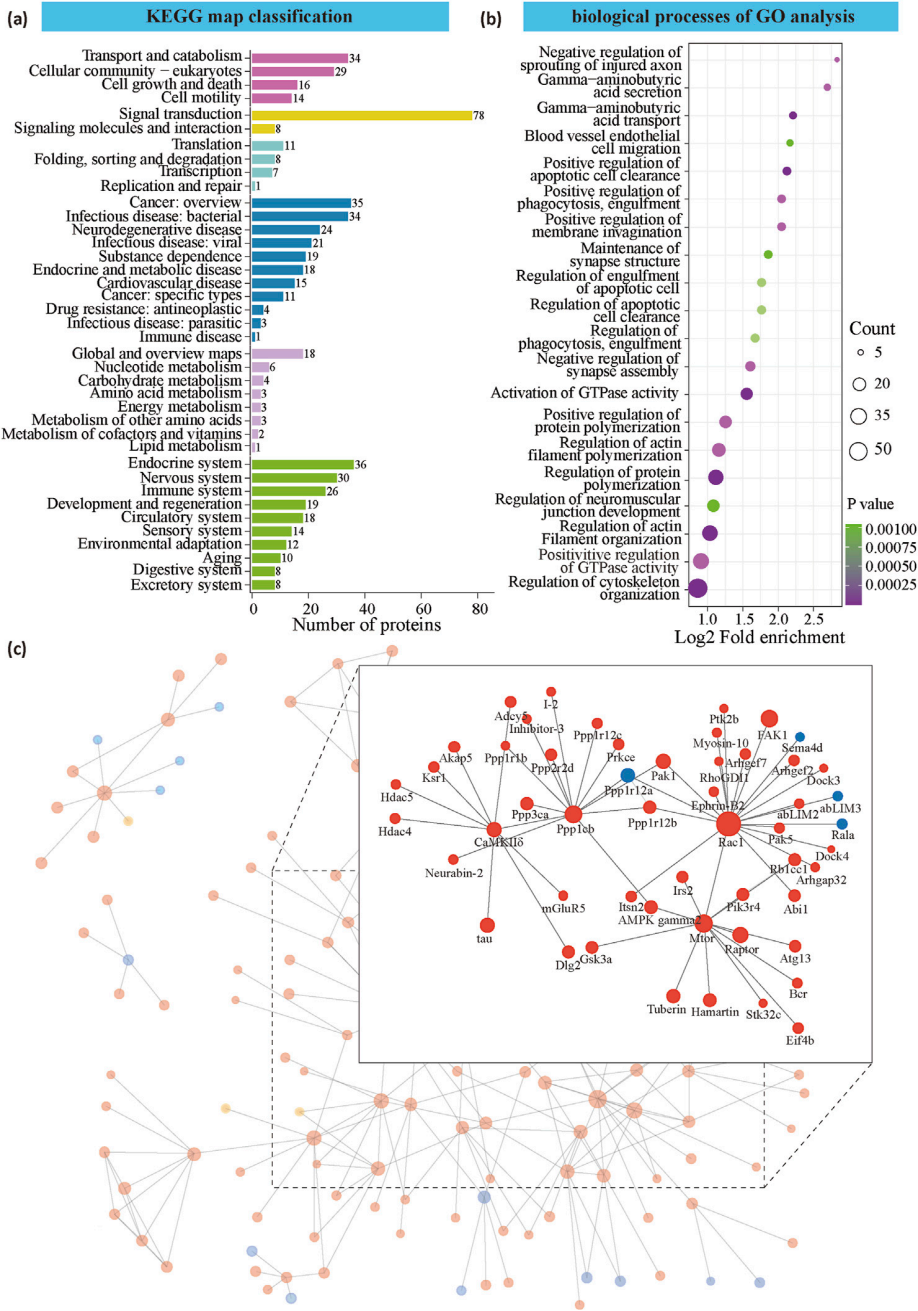
Functional analysis of differential expression protein between dominant and subordinate. **(a)** Cluster of orthologous groups of proteins by KEGG map classification. **(b)** Gene ontology analysis of gene networks associated with biological processes. **(c)** The top 50 most closely interacting proteins map the protein interaction network. Blue is the downregulated modified protein, red is the up-regulated modified protein, and yellow is the modified protein that also contains the up-regulated modification site.

To functionally categorize the significantly differentially expressed phosphoproteins, we performed KEGG and GO analysis. KEGG analysis revealed that signal transduction was the most significantly altered pathway, with 78 phosphoproteins showing changes, suggesting distinct signal transduction mechanisms in the NAc between dominant and subordinate rats (Figure 3A). Notably, phosphoproteins associated with substance dependence were also enriched in disease-related pathways, indicating that social hierarchy may play a role in modulating vulnerability to drug addiction. Further analysis of organismal systems revealed that the significant phosphoproteins were primarily enriched in the endocrine and nervous systems. Given that subordinate mice likely experience heightened stress, it is reasonable to infer that the differential proteins are enriched in the endocrine system, reflecting the physiological response to stress and its potential influence on behavior and drug reinforcement.

GO analysis revealed that the differentially phosphorylated proteins were significantly enriched in processes related to cytoskeleton regulation, including the regulation of cytoskeleton organization, positive regulation of GTPase activity, regulation of actin filament organization, and regulation of actin filament polymerization (Figure 3B). Additionally, proteins involved in synapse assembly (e.g., Fnbp1l, Ptk2b, and Gsk3a) and synaptic structure (e.g., Shank3, Pclo, and Erc2) were also enriched (Figure 3C). These findings collectively suggest that the differentially expressed phosphorylated proteins influence cellular and dendritic morphology, as well as synaptic remodeling, which may serve as a link between social hierarchy and drug reinforcement. This highlights the potential role of synaptic plasticity and cytoskeletal dynamics in mediating the behavioral effects associated with social rank and substance use.

To identify key hub proteins, we used IPA software to construct and visualize a protein interaction network. The analysis revealed that Ras-related C3 botulinum toxin substrate 1 (Rac1), Serine/threonine-protein kinase mTOR (Mtor), Serine/threonine-protein phosphatase PP1-beta catalytic subunit (Ppp1cb), and Calcium/calmodulin-dependent protein kinase type II subunit delta (CaMKIIδ) were key hub proteins (Figure 3C). Rac1, a small GTPase from the Rho family, is essential for actin cytoskeleton remodeling. Mtor, a serine/threonine kinase, plays a central role in actin cytoskeleton regulation. Ppp1cb is an isoform of protein phosphatase 1 (PP1) and may act as a negative regulator of learning and memory. CaMKIIδ, a calcium/calmodulin-dependent protein kinase, is critically involved in synaptic plasticity and memory processes. Mtor, Ppp1cb, and CaMKIIδ regulate protein phosphorylation and signal transduction, while Rac1 and Mtor are key regulators of cytoskeleton remodeling.

### Phosphorylation motifs and upstream kinases are characterized by social hierarchy

**FIGURE 4:**
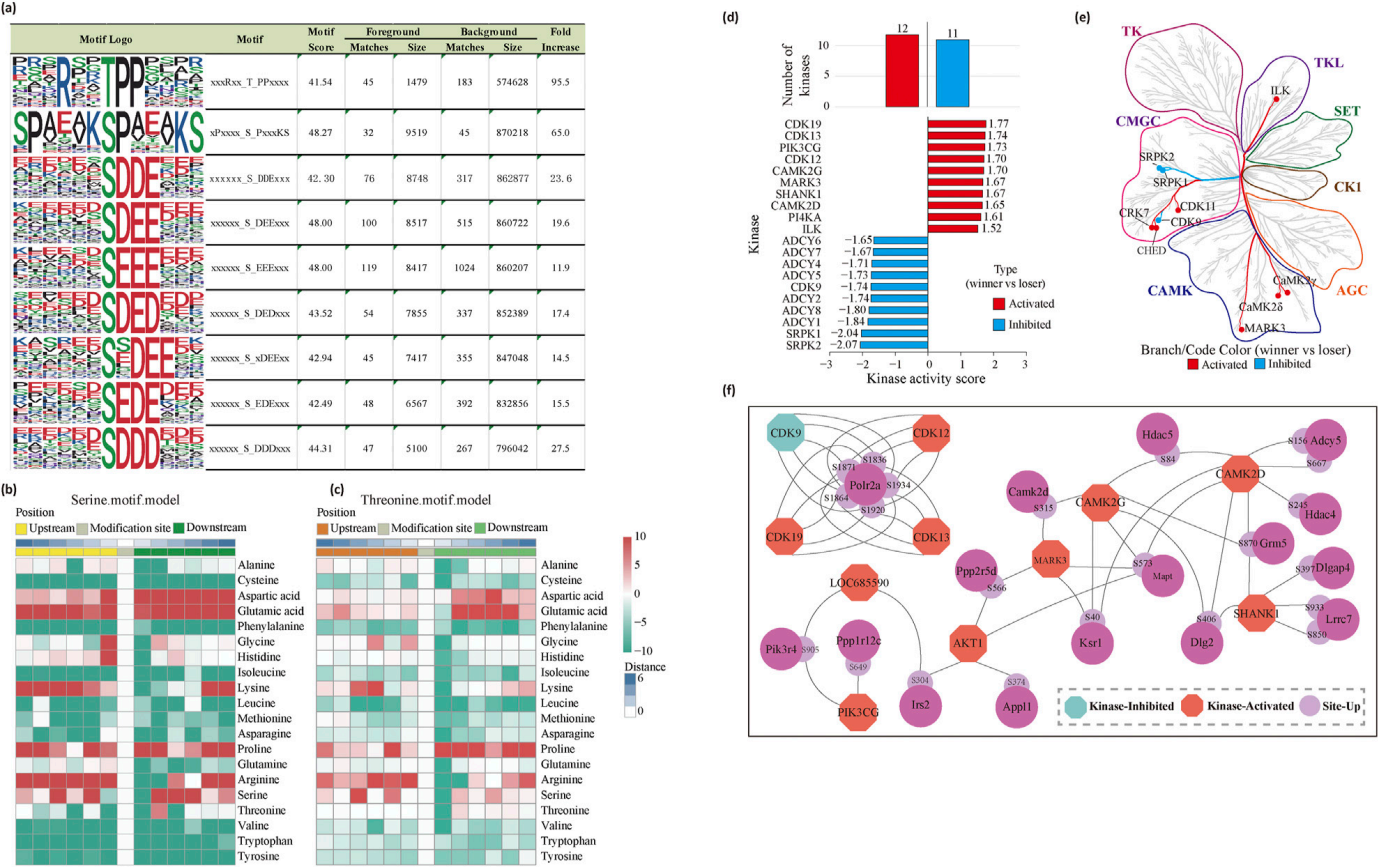
Phosphorylation motifs analysis. **(a)** Specific motif logo of tube test model **(b, c)** The heatmap, including serine. motif.model **(b)** and threonine. motif.model **(c)** shows the degree of variation in the frequency of amino acids near phosphorylation modification sites. **(d)** Prediction of kinase activity. **(e)** Canonical phosphosites on kinases mapped onto the kinome phylogenetic tree. **(f)** Protein interaction network of PHOTON predicted cycling active kinases.

We analyzed the sequences of the phosphorylated peptides to identify phosphorylation patterns through motif analysis. The analysis revealed eight serine motifs and one threonine motif, each exhibiting a motif score greater than 40 and a fold enrichment above 10 (Figure 4A). For serine phosphorylation sites, the conserved amino acids surrounding the phospho-sites included aspartic acid (D), glutamic acid (E), proline (P), arginine (R), and serine (S). Notably, aspartic acid, glutamic acid, and proline were frequently observed in the −1 to −6 (upstream) and 1 to 6 (downstream) positions relative to the phosphorylated residues (Figure 4B). A similar pattern was found for the threonine motif, although for threonine, aspartic acid, glutamic acid, and proline were predominantly enriched only at the downstream positions (1–6) (Figure 4C). These findings suggest that specific amino acid patterns surrounding phosphorylation sites are conserved and may play a role in the regulation of phosphorylation events, which could be critical for understanding the mechanisms underlying social hierarchy and drug reinforcement.

To predict kinase activity and identify key kinases involved in phosphorylation regulation in dominant rats, we conducted kinase activity predictions using several tools and databases, including GPS5.0 for kinase-substrate regulation predictions, IEKPD2.0 for identifying kinase sequences, and GSEA to assess kinase activity. This analysis revealed 12 activated and 11 inhibited kinases in the dominant rats. We further focused on the top 10 phosphokinases, ranked by their activation and inhibition scores (Figure 4D). Our findings showed that these kinases span multiple major kinase families, with a particular enrichment in the CMGC and CAMK families (Figure 4E). Notably, some of the identified kinases are known to be involved in drug addiction. For instance, CaMKIIδ has been reported to regulate phosphorylation sites that are implicated in addiction-related pathways, including targets such as mGluR5, HDAC4, and HDAC5 (Lobo et al., 2015) (Figure 4F). These results suggest that the kinases involved in social hierarchy may also play a critical role in modulating drug addiction-related pathways.

### The phosphorylation of HADC4 is significantly higher in dominants

**FIGURE 5:**
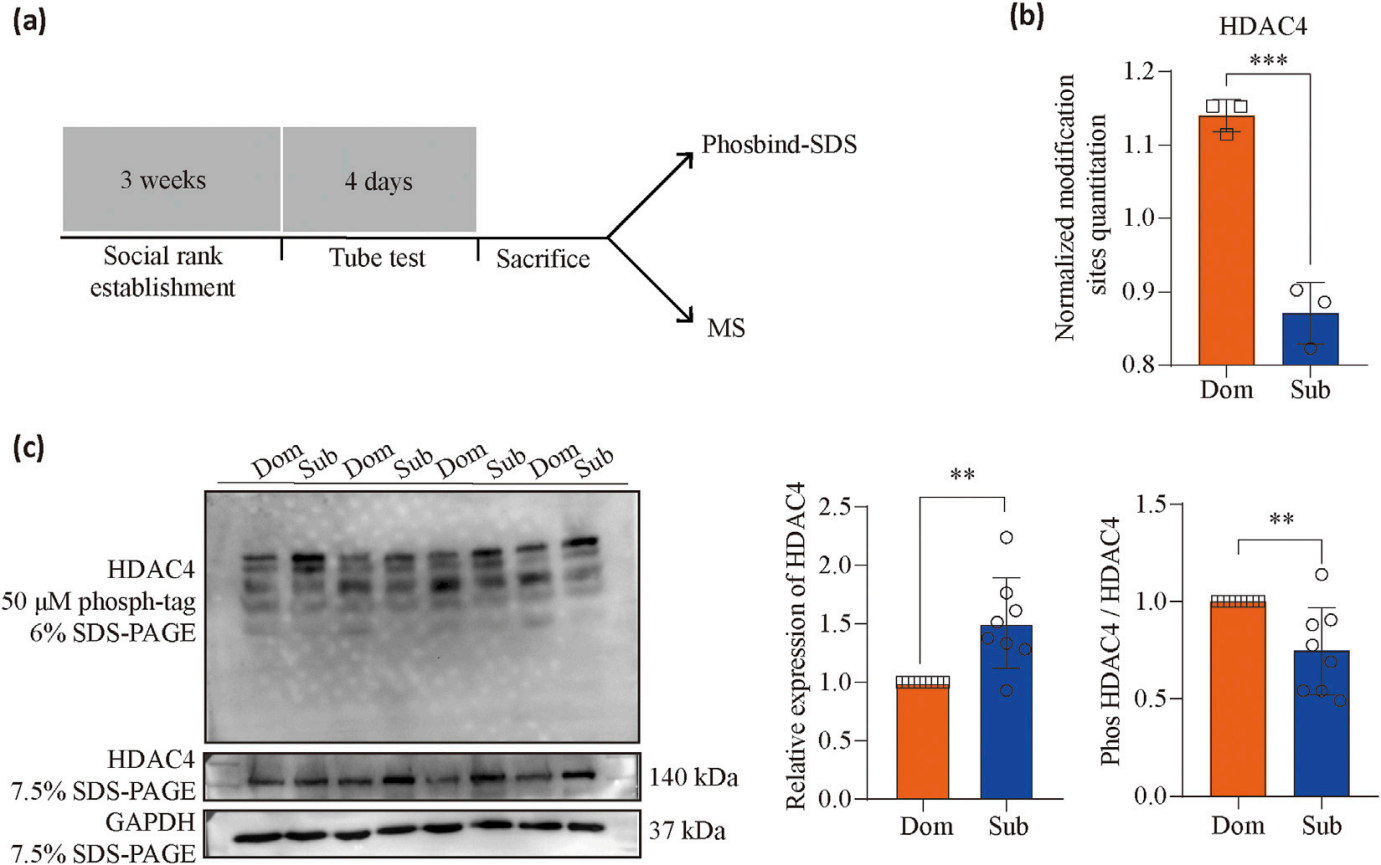
MS-based analysis and immunoblots for key proteins phosphorylation. **(a)** Timeline of the behavioral paradigms followed. **(b)** MS-based analysis for HDAC4 (*n* = 3, Unpaired t-test, *t* (4) = 9.814, *P* = 0.0006) phosphorylation. **(c)** Quantitative analysis of immunoblots using specific antibodies for HDAC4 (*n* = 8, Unpaired t-test, t (14) = 3.708, P = 0.0023) and HDAC4 phosphorylated protein motifs (*n* = 8, Unpaired t-test, *t* (14) = 3.211, *P* = 0.0063). **P < 0.01, ***P < 0.001. Data represent means ± SD. Dom, dominants. Sub, subordinates.

From the PPI and kinase prediction analyses, CaMKIIδ kinase and its downstream proteins, such as mGluR5 and HDAC4, emerged as key mediators of the drug reinforcement differences associated with social hierarchy. To validate the phosphoproteomics results, we utilized the phosbind-SDS technique, which measures the overall phosphorylation levels of proteins rather than site-specific modifications. First, we analyzed the overall phosphorylation levels of CaMKIIδ downstream proteins identified in the mass spectrometry data and selected proteins with significant changes for phosbind-SDS detection. Mass spectrometry revealed that the overall phosphorylation level of HDAC4 was significantly higher in the dominant group compared to the subordinate group (Unpaired *t*-test, *t* (4) = 9.814, *P* = 0.0006) (Figure 5B). Phosbind-SDS detection corroborated these findings, showing that certain phosphorylation sites on HDAC4 exhibited higher phosphorylation levels in the dominant group, while others displayed lower levels. Importantly, the cumulative phosphorylation percentage was significantly higher in the dominant group than in the subordinate group (Unpaired *t*-test, *t* (14) = 3.211, *P* = 0.0063) (Figure 5C), aligning with the phosphorylation mass spectrometry results. These findings highlight the role of HDAC4 phosphorylation in the molecular mechanisms underlying social hierarchy and drug reinforcement.

### Behavioral alteration of social hierarchy modulates protein phosphorylation in the NAc and drug reinforcement

**FIGURE 6:**
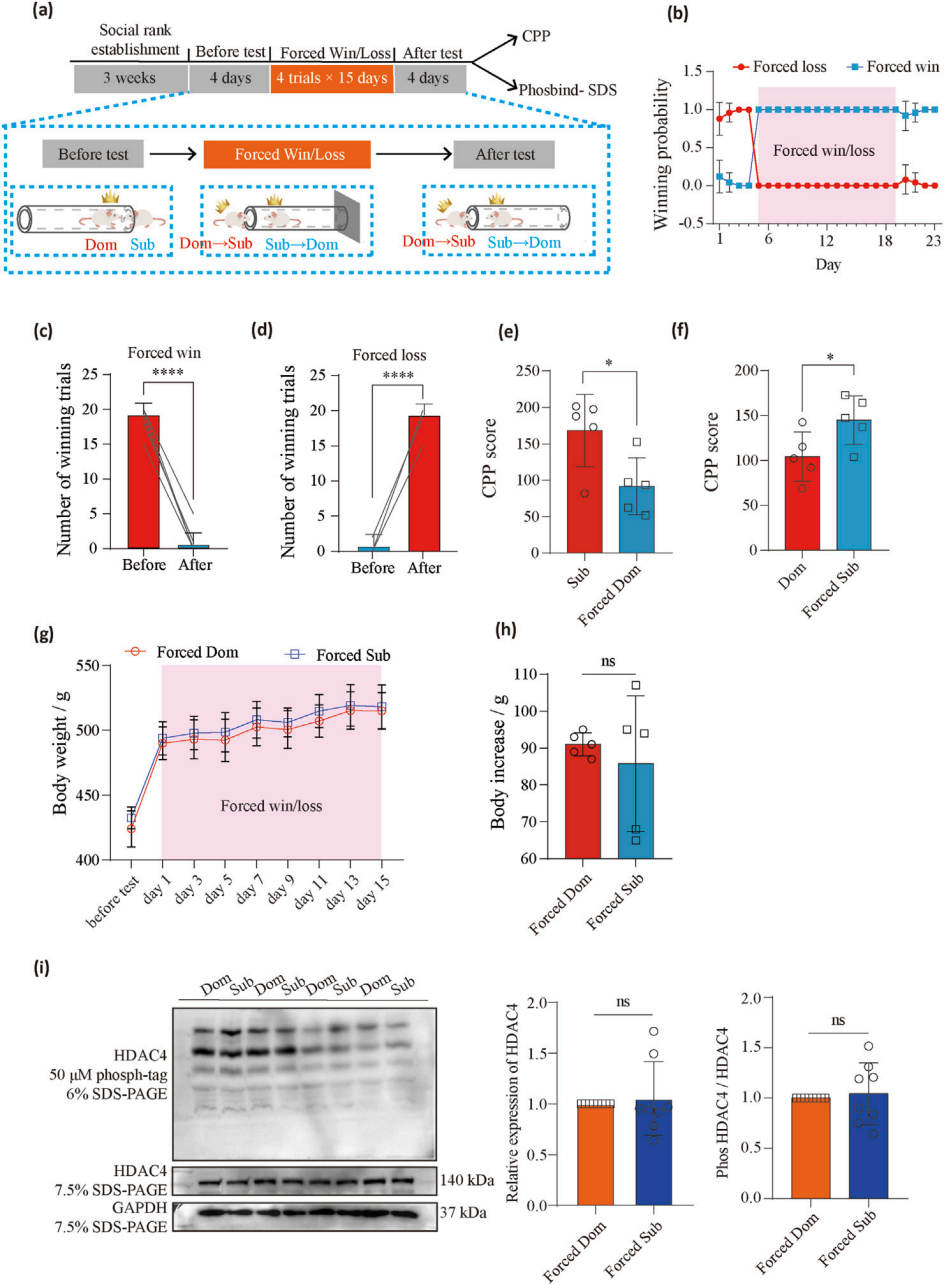
Forced win/loss behavioral training reversed meth preference. **(a)** Timeline of the behavioral paradigms followed. **(b)** The change in winning probability across sessions in rats (*n* = 10) transitioning from subordinates to dominants or dominants to subordinates after the forced win/loss training in the tube test. **(c)** The total number of win trials before and after forced win (*n* = 10 pairs, paired *t*-test, *t* (9) = 27.76, *P* < 0.0001). **(d)** The total number of win trials before and after forced loss (*n* = 10 pairs, paired *t*-test, *t* (9) = 27.76, *P* < 0.0001). **(e)** Comparison of CPP scores between the subordinate (*n* = 5) and forced dominant (*n* = 5) groups (Unpaired t-test, *t* (8) = 2.715, *P* = 0.0264). **(f)** Comparison of CPP scores between the dominant (*n* = 5) and forced subordinate (*n* = 5) groups (Unpaired t-test, *t* (8) = 2.371, *P* = 0.0452). **(g)** The change of body weight during forced win/loss. **(h)** Comparison of body increase between forced dominants (*n* = 5) and forced subordinates (*n* = 5) (paired *t*-test, *t* (4) = 0.6236, *P* = 0.5503) after forced win/loss. **(i)** Quantitative analysis of immunoblots using specific antibodies for HDAC4 (*n* = 8, Unpaired t-test, *t* (14) = 0.4223, *P* = 0.6792) and HDAC4 phosphorylated protein motifs (*n* = 8, Unpaired t-test, *t* (14) = 0.4105, *P* = 0.6876). ns (not significant), *P < 0.05, ****P < 0.0001. Data represent means ± SD. Dom, dominants. Sub, subordinates.

To explore whether altering social hierarchy can change protein phosphorylation status and drug reinforcement, we first reversed the social hierarchy of rats through behavioral training. After establishing a stable social hierarchy among the rats, we used the tube test to block the retreat of the subordinate rats, forcing them to advance until they pushed the dominant rats out of the tube (Fan et al., 2023). After 15 days of this “forced winning” training, we conducted a standard 4-day tube test. We found that the original subordinate rats were now able to win on their own abilities, effectively becoming dominant rats (Figures 6B, C). We labeled these rats as “forced dominants,” while those that lost after training were called “forced subordinates.”

The rats were then divided into two groups: one group underwent behavioral testing to observe how social hierarchy changes affected drug reinforcement, while the other group was subjected to phosbind-SDS to assess the overall phosphorylation level of HDAC4 in the NAc region, examining how social hierarchy changes impacted phosphorylation levels. We found that the forced dominants had significantly reduced methamphetamine (METH) conditioned place preference (CPP) scores (Unpaired *t*-test, *t* (8) = 2.715, *P* = 0.0264) (Figure 6E), while the forced subordinates showed a significant increase in their CPP scores (Unpaired *t*-test, *t* (8) = 2.371, *P* = 0.0452) (Figure 6F). We recorded the body weight at the time of before test and forced win/loss. There were no significantly different in the body weight and body increase between the forced dominants and forced subordinates (paired *t*-test, *t* (4) = 0.6236, *P* = 0.5503) (Figures 6G, H). Additionally, the forced win condition reversed the phosphorylation of HDAC4 in the NAc of the subordinate rats (Unpaired *t*-test, *t* (14) = 0.4105, *P* = 0.6876) (Figure 6I). These findings suggest that changes in social hierarchy can reorganize protein phosphorylation and modulate METH reinforcement, highlighting the dynamic interplay between social rank and drug reinforcement behaviors.

## Discussion

In the present study, we demonstrate that social hierarchy significantly influences the phosphoproteomic profiles of the nucleus accumbens (NAc). Notably, the altered phosphoproteins were enriched in pathways related to signal transduction and synaptic remodeling. These findings suggest that the molecular underpinnings of social rank may modulate cellular processes that are also critical for drug reinforcement. Through motif analysis, we identified specific phosphorylation patterns associated with social hierarchy, and we predicted the upstream kinases responsible for modulating these phosphorylation events. Using Phosbind-SDS detection, we validated our phosphoproteomics findings, focusing on proteins such as HDAC4, which showed significant changes in phosphorylation levels between dominant and subordinate rats. Lastly, we employed behavioral training to manipulate social hierarchy and observed corresponding changes in drug reinforcement behaviors and HDAC4 phosphorylation. These results underscore the plasticity of both molecular signaling and behavior in response to changes in social rank, highlighting the role of phosphorylation in modulating addiction-related processes.

Although phosphoproteomics has been widely studied in the context of drug addiction, no comprehensive phosphoproteomic analysis has been conducted specifically in relation to social hierarchy until now. Our study is the first to provide valuable data showing how the social environment influences protein phosphorylation and addiction-like behaviors. Notably, we found that social hierarchy induces differential phosphorylation of proteins that are enriched in the substance dependence pathway. This observation aligns with previous studies demonstrating that addiction vulnerability is modulated by social status across species, including rodents, non-human primates, and humans (Bardo et al., 2013b; Lo and Cheng, 2012). Moreover, Gene Ontology (GO) analysis revealed that the differentially phosphorylated proteins are primarily involved in cytoskeleton organization and the regulation of GTPase activity. These findings are consistent with several studies indicating that cytoskeleton-related proteins play a crucial role in learning and the formation of drug-related memories (Salery et al., 2017; Shah and Rossie, 2018). Furthermore, cytoskeletal proteins and their regulatory mechanisms are emerging as promising therapeutic targets for drug addiction treatment (Pandey and Miller, 2024). Our findings provide a deeper understanding of the molecular interplay between social hierarchy and addiction, offering potential insights into novel avenues for addiction interventions.

Both protein-protein interaction (PPI) analysis and kinase prediction suggest that CaMKIIδ serves as a central hub in the differential phosphoproteins between dominant and subordinate rats. While CaMKIIα has been well-established as a key protein in synaptic plasticity regulation and drug addiction (Yasuda et al., 2022), the role of CaMKIIδ in addiction remains unexplored. CaMKIIδ, a subunit of the CaMKII complex, is known to promote neurotransmitter release, regulate neuronal excitability, and enhance synaptic plasticity (Sun et al., 2022). Our findings suggest that CaMKIIδ may play a critical role in both social hierarchy and drug reinforcement processes. Interestingly, many of the downstream proteins phosphorylated by CaMKIIδ are also implicated in drug addiction. For example, mGluR5, a key player in modulating rapid changes in synaptic transmission and plasticity, has been identified as a crucial molecular target involved in all phases of drug addiction (Abd-Elrahman and Ferguson, 2022; Murray et al., 2021; Wang et al., 2021). HDAC4, a protein that regulates synaptic plasticity and plasticity-related genes (Puma et al., 2023), has been shown to enhance compulsive cocaine self-administration (Edmund et al., 2017; Wang et al., 2010). Additionally, adenylate cyclases (ADCYs), involved in spatial learning and long-term potentiation (LTP) (Devasani and Yao, 2022), have been linked to the polygenic etiology of alcoholism (Procopio et al., 2013). These findings highlight the potential of CaMKIIδ as a key molecular mediator that bridges social status and addiction-related behaviors, underscoring its importance as a therapeutic target in addiction treatment.

We further identified HDAC4-S245 as a novel downstream target of CaMKIIδ, representing a previously unrecognized phosphorylation site involved in social hierarchy and drug addiction. Our findings suggest that behavioral training can modulate not only social rank and HDAC4 phosphorylation but also drug reinforcement. This is consistent with the role of HDAC4 as a critical epigenetic regulator of gene expression, emphasizing its involvement in regulating various functions, including synaptic plasticity and addiction-related processes. By controlling HDAC4 activity, the brain could dynamically adjust responses to social and drug-related stimuli. However, one limitation of our study is the lack of direct verification of the S245 phosphorylation site specifically in the context of social hierarchy and drug reinforcement. The identification of HDAC4-S245 as a target of CaMKIIδ is a crucial step, but further validation experiments, such as using phospho-specific antibodies or site-directed mutagenesis, will be necessary to confirm its functional significance. Future studies will aim to explore the functional consequences of mutating HDAC4-S245, potentially utilizing behavioral assays to assess changes in social hierarchy, drug reinforcement, and associated molecular pathways.

In conclusion, our study underscores the critical role of protein phosphorylation in regulating social hierarchy and its impact on drug reinforcement. The shift in social rank corresponded with changes in drug reinforcement. These findings highlight that social status is not only a behavioral trait but also a key determinant of the neurobiological mechanisms underlying addiction, with protein phosphorylation serving as a crucial modulator of these processes. This research contributes to a deeper understanding of how social factors influence addiction and suggests that targeting phosphorylation pathways may provide novel strategies for addressing addiction-related behaviors.

## Data Availability

The original contributions presented in the study are publicly available. This data can be found here: ProteomeXchange Consortium, accession number PXD062735 (https://www.ebi.ac.uk/pride/archive/projects/PXD062735).

## References

[B1] Abd-Elrahman K. S. Ferguson S. S. G. (2022). Noncanonical metabotropic glutamate receptor 5 signaling in alzheimer's disease. Annu. Rev. Pharmacol. Toxicol. 62, 235–254. 10.1146/annurev-pharmtox-021821-091747 34516293

[B2] Bardo M. T. Neisewander J. L. Kelly T. H. (2013a). Individual differences and social influences on the neurobehavioral pharmacology of abused drugs. Pharmacol. Rev. 65 (1), 255–290. 10.1124/pr.111.005124 23343975 PMC3565917

[B3] Bardo M. T. Neisewander J. L. Kelly T. H. (2013b). Individual differences and social influences on the neurobehavioral pharmacology of abused drugs. Pharmacol. Rev. 65 (1), 255–290. 10.1124/pr.111.005124 23343975 PMC3565917

[B4] Bernstein D. L. Lewandowski S. I. Besada C. Place D. España R. A. Mortensen O. V. (2024). Inactivation of ERK1/2 signaling in dopaminergic neurons by map kinase phosphatase MKP3 regulates dopamine signaling and motivation for cocaine. J. Neurosci. Official J. Soc. Neurosci. 44 (5), e0727232023. 10.1523/JNEUROSCI.0727-23.2023 38296649 PMC10860627

[B5] Bilbrough T. Piemontese E. Seitz O. (2022). Dissecting the role of protein phosphorylation: a chemical biology toolbox. Chem. Soc. Rev. 51 (13), 5691–5730. 10.1039/d1cs00991e 35726784

[B6] Cadet J. L. Brannock C. Jayanthi S. Krasnova I. N. (2015). Transcriptional and epigenetic substrates of methamphetamine addiction and withdrawal: evidence from a long-access self-administration model in the rat. Mol. Neurobiol. 51 (2), 696–717. 10.1007/s12035-014-8776-8 24939695 PMC4359351

[B7] Castro D. C. Bruchas M. R. (2019). A motivational and neuropeptidergic hub: anatomical and functional diversity within the nucleus accumbens shell. Neuron 102 (3), 529–552. 10.1016/j.neuron.2019.03.003 31071288 PMC6528838

[B8] Choi T. Jeon H. Jeong S. Kim E. Kim J. Jeong Y. (2024). Distinct prefrontal projection activity and transcriptional state conversely orchestrate social competition and hierarchy. Neuron 112 (4), 611–627.e8. 10.1016/j.neuron.2023.11.012 38086372

[B9] Devasani K. Yao Y. (2022). Expression and functions of adenylyl cyclases in the CNS. Fluids Barriers CNS 19 (1), 23. 10.1186/s12987-022-00322-2 35307032 PMC8935726

[B10] Duarte M. L. Devi L. A. (2020). Post-translational modifications of opioid receptors. Trends Neurosci. 43 (6), 417–432. 10.1016/j.tins.2020.03.011 32459993 PMC7323054

[B11] Edmund A. Griffin Jr P. A. M. R. Mercado P. Kempadoo K. A. Stephenson S. Colnaghi L. (2017). Prior alcohol use enhances vulnerability to compulsive cocaine self-administration by promoting degradation of HDAC4 and HDAC5. Sci. Adv. 3 (11), e1701682. 10.1126/sciadv.1701682 29109977 PMC5665598

[B12] Fan Z. Chang J. Liang Y. Zhu H. Zhang C. Zheng D. (2023). Neural mechanism underlying depressive-like state associated with social status loss. Cell 186 (3), 560–576.e17. 10.1016/j.cell.2022.12.033 36693374

[B13] Fan Z. Zhu H. Zhou T. Wang S. Wu Y. Hu H. (2019). Using the tube test to measure social hierarchy in mice. Nat. Protoc. 14 (3), 819–831. 10.1038/s41596-018-0116-4 30770887

[B14] Gilmour K. M. Craig P. M. Dhillon R. S. Lau G. Y. Richards J. G. (2017). Regulation of energy metabolism during social interactions in rainbow trout: a role for AMP-activated protein kinase. Am. J. Physiology. Regul. Integr. Comp. Physiology 313 (5), R549-R559–R559. 10.1152/ajpregu.00341.2016 28768660 PMC5792151

[B15] Gould R. W. Czoty P. W. Porrino L. J. Nader M. A. (2017). Social status in monkeys: effects of social confrontation on brain function and cocaine self-administration. Neuropsychopharmacol. Official Publ. Am. Coll. Neuropsychopharmacol. 42 (5), 1093–1102. 10.1038/npp.2016.285 28025974 PMC5506801

[B16] Kibaly C. Kam A. Y. F. Loh H. H. Law P. (2016). Naltrexone facilitates learning and delays extinction by increasing AMPA receptor phosphorylation and membrane insertion. Biol. Psychiatry 79 (11), 906–916. 10.1016/j.biopsych.2015.04.019 26049209 PMC4630208

[B17] Kim S. Sohn S. Choe E. S. (2022). Phosphorylation of GluA1-ser831 by CaMKII activation in the caudate and putamen is required for behavioral sensitization after challenge nicotine in rats. Int. J. Neuropsychopharmacol. 25 (8), 678–687. 10.1093/ijnp/pyac034 35678163 PMC9380710

[B18] Koob G. F. Volkow N. D. (2016). Neurobiology of addiction: a neurocircuitry analysis. Lancet 3 (8), 760–773. 10.1016/S2215-0366(16)00104-8 27475769 PMC6135092

[B19] Larrieu T. Cherix A. Duque A. Rodrigues J. Lei H. Gruetter R. (2017). Hierarchical status predicts behavioral vulnerability and nucleus accumbens metabolic profile following chronic social defeat stress. Curr. Biol. 27 (14), 2202–2210. 10.1016/j.cub.2017.06.027 28712571

[B20] Lee A. M. Messing R. O. (2008). Protein kinases and addiction. Ann. N. Y. Acad. Sci. 1141, 22–57. 10.1196/annals.1441.022 18991950 PMC3050040

[B21] Lo C. C. Cheng T. C. (2012). Discrimination's role in minority groups' rates of substance-use disorder. Am. J. Addict. 21 (2), 150–156. 10.1111/j.1521-0391.2011.00205.x 22332859

[B22] Lobo D. S. S. Aleksandrova L. Knight J. Casey D. M. El-Guebaly N. Nobrega J. N. (2015). Addiction-related genes in gambling disorders: new insights from parallel human and pre-clinical models. Mol. Psychiatry 20 (8), 1002–1010. 10.1038/mp.2014.113 25266122

[B23] Milewski T. M. Lee W. Champagne F. A. Curley J. P. (2022). Behavioural and physiological plasticity in social hierarchies. Philosophical Trans. R. Soc. Lond. Ser. B, Biol. Sci. 377 (1845), 20200443. 10.1098/rstb.2020.0443 35000436 PMC8743892

[B24] Morgan D. Grant K. Gage H. Mach R. Kaplan J. Prioleau O. (2002). Social dominance in monkeys: dopamine d2 receptors and cocaine self-administration. Nat. Neurosci. 5 (2), 169–174. 10.1038/nn798 11802171

[B25] Murray C. H. Christian D. T. Milovanovic M. Loweth J. A. Hwang E. Caccamise A. J. (2021). mGlu5 function in the nucleus accumbens core during the incubation of methamphetamine craving. Neuropharmacology 186, 108452. 10.1016/j.neuropharm.2021.108452 33444640 PMC8440156

[B26] Nader M. A. Czoty P. W. Gould R. W. Riddick N. V. (2008). Review. Positron emission tomography imaging studies of dopamine receptors in primate models of addiction. Philosophical Trans. R. Soc. Lond. Ser. B, Biol. Sci. 363 (1507), 3223–3232. 10.1098/rstb.2008.0092 18640923 PMC2607324

[B27] Nestler E. J. Landsman D. (2001). Learning about addiction from the genome. Nature 409 (6822), 834–835. 10.1038/35057015 11237002

[B28] Pandey S. Miller C. A. (2024). IUPHAR-review: targeting the cytoskeleton as a therapeutic approach to substance use disorders. Pharmacol. Res. 202, 107143. 10.1016/j.phrs.2024.107143 38499081 PMC11034636

[B29] Procopio D. O. Saba L. M. Walter H. Lesch O. Skala K. Schlaff G. (2013). Genetic markers of comorbid depression and alcoholism in women. Alcohol. Clin. Exp. Res. 37 (6), 896–904. 10.1111/acer.12060 23278386 PMC3620932

[B30] Puma D. D. L. Colussi C. Bandiera B. Puliatti G. Rinaudo M. Cocco S. (2023). Interleukin 1β triggers synaptic and memory deficits in Herpes simplex virus type-1-infected mice by downregulating the expression of synaptic plasticity-related genes via the epigenetic MeCP2/HDAC4 complex. Cell. Mol. Life Sci. CMLS 80 (6), 172. 10.1007/s00018-023-04817-5 37261502 PMC10234878

[B31] Salery M. Dos Santos M. Saint-Jour E. Moumné L. Pagès C. Kappès V. (2017). Activity-regulated cytoskeleton-associated protein accumulates in the nucleus in response to cocaine and acts as a brake on chromatin remodeling and long-term behavioral alterations. Biol. Psychiatry, 81(7), 573–584. 10.1016/j.biopsych.2016.05.025 27567310

[B32] Sapolsky R. M. (2005). The influence of social hierarchy on primate health. Science 308 (5722), 648–652. 10.1126/science.1106477 15860617

[B33] Schall T. A. Wright W. J. Dong Y. (2021). Nucleus accumbens fast-spiking interneurons in motivational and addictive behaviors. Mol. Psychiatry 26 (1), 234–246. 10.1038/s41380-020-0683-y 32071384 PMC7431371

[B34] Shah K. Rossie S. (2018). Tale of the good and the bad Cdk5: remodeling of the actin cytoskeleton in the brain. Mol. Neurobiol. 55 (4), 3426–3438. 10.1007/s12035-017-0525-3 28502042 PMC6370010

[B35] Sun L. Chen M. Wang H. Dong J. Zhao L. Peng R. (2022). CaMKIIδ promotes synaptic plasticity under terahertz wave radiation by activation of the NF-κB pathway. J. Phys. Chem. Lett. 13 (25), 5925–5931. 10.1021/acs.jpclett.2c00775 35731851

[B36] Swendsen J. Le Moal M. (2011). Individual vulnerability to addiction. Ann. N. Y. Acad. Sci. 1216, 73–85. 10.1111/j.1749-6632.2010.05894.x 21272012

[B37] Torregrossa M. M. MacDonald M. Stone K. L. Lam T. T. Nairn A. C. Taylor J. R. (2019). Phosphoproteomic analysis of cocaine memory extinction and reconsolidation in the nucleus accumbens. Psychopharmacology 236 (1), 531–543. 10.1007/s00213-018-5071-9 30411139 PMC6374162

[B38] Volkow N. D. Michaelides M. Baler R. (2019). The neuroscience of drug reward and addiction. Physiol. Rev. 99 (4), 2115–2140. 10.1152/physrev.00014.2018 31507244 PMC6890985

[B39] Wang L. Lv Z. Hu Z. Sheng J. Hui B. Sun J. (2010). Chronic cocaine-induced H3 acetylation and transcriptional activation of CaMKIIalpha in the nucleus accumbens is critical for motivation for drug reinforcement. Neuropsychopharmacol. Official Publ. Am. Coll. Neuropsychopharmacol. 35 (4), 913–928. 10.1038/npp.2009.193 20010550 PMC3055366

[B40] Wang Y. Guo R. Chen B. Rahman T. Cai L. Li Y. (2021). Cocaine-induced neural adaptations in the lateral hypothalamic melanin-concentrating hormone neurons and the role in regulating rapid eye movement sleep after withdrawal. Mol. Psychiatry 26 (7), 3152–3168. 10.1038/s41380-020-00921-1 33093653 PMC8060355

[B41] Yap J. J. Chartoff E. H. Holly E. N. Potter D. N. Jr W. A. C. Miczek K. A. (2015). Social defeat stress-induced sensitization and escalated cocaine self-administration: the role of ERK signaling in the rat ventral tegmental area. Psychopharmacology 232 (9), 1555–1569. 10.1007/s00213-014-3796-7 25373870 PMC4397167

[B42] Yasuda R. Hayashi Y. Hell J. W. (2022). CaMKII: a central molecular organizer of synaptic plasticity, learning and memory. Nat. Rev. Neurosci. 23 (11), 666–682. 10.1038/s41583-022-00624-2 36056211

[B43] Yoshida M. Hasegawa S. Taniguchi M. Mouri A. Suzuki C. Yoshimi A. (2022). Memantine ameliorates the impairment of social behaviors induced by a single social defeat stress as juveniles. Neuropharmacology 217, 109208. 10.1016/j.neuropharm.2022.109208 35926580

