# Correction: Social hierarchy modulates drug reinforcement and protein phosphorylation in the nucleus accumbens

**DOI:** 10.3389/fphar.2026.1895604

**Published:** 2026-07-02

**Authors:** Liang Xu, Ruiyi Zhou, Jiafeng Zhong, Yina Huang, Yingjie Zhu, Wei Xu

**Affiliations:** 1 West China School of Public Health and West China Fourth Hospital, Sichuan University, Chengdu, China; 2 Faculty of Life and Health Sciences, Shenzhen University of Advanced Technology, Shenzhen, China; 3 Shenzhen Key Laboratory of Drug Addiction, Shenzhen Neher Neural Plasticity Laboratory, The Brain Cognition and Brain Disease Institute, Shenzhen Institute of Advanced Technology, Chinese Academy of Sciences, Shenzhen, China; 4 University of Chinese Academy of Sciences, Beijing, China; 5 CAS Key Laboratory of Brain Connectome and Manipulation, The Brain Cognition and Brain Disease Institute, Shenzhen Institute of Advanced Technology, Chinese Academy of Sciences, Shenzhen, China

**Keywords:** social hierarchy, drug reinforcement, phosphoproteomics, drug addiction, HDAC4

In the published article, there was a mistake in the Materials and methods. A correction has been made to the section **Materials and methods**, Animals, Paragraph 1. The subsection is now renamed as “Animals and Drugs”.

The paragraph was incorrectly written as:

“Male Sprague-Dawley rats, aged 5–6 weeks, were obtained from Charles River (Beijing, China) for this study. The animals were housed under controlled conditions at a temperature of 22 °C–25 °C with a 12-hour light–dark cycle. All experimental procedures and animal care protocols were approved by the Animal Care and Use Committee of the Shenzhen Institute of Advanced Technology (SIAT), Chinese Academy of Sciences (CAS).”

The correct statement is “Male Sprague-Dawley rats, aged 5–6 weeks, were obtained from Charles River (Beijing, China) for this study. The animals were housed under controlled conditions at a temperature of 22 °C–25 °C with a 12-hour light–dark cycle. All experimental procedures and animal care protocols were approved by the Animal Care and Use Committee of the Shenzhen Institute of Advanced Technology (SIAT), Chinese Academy of Sciences (CAS). The methamphetamine (>99% purity, PE-0846) used in this study was purchased from Shanghai Yuansi Standard Science and Technology Co., Ltd.”

The original article has been updated.

