# Supplementary figures and images for: Social hierarchy modulates drug reinforcement and protein phosphorylation in the nucleus accumbens
Source: Front Pharmacol. 2025 Apr 11;16:1537131. doi: 10.3389/fphar.2025.1537131 (PMC12022441; doi:10.3389/fphar.2025.1537131)

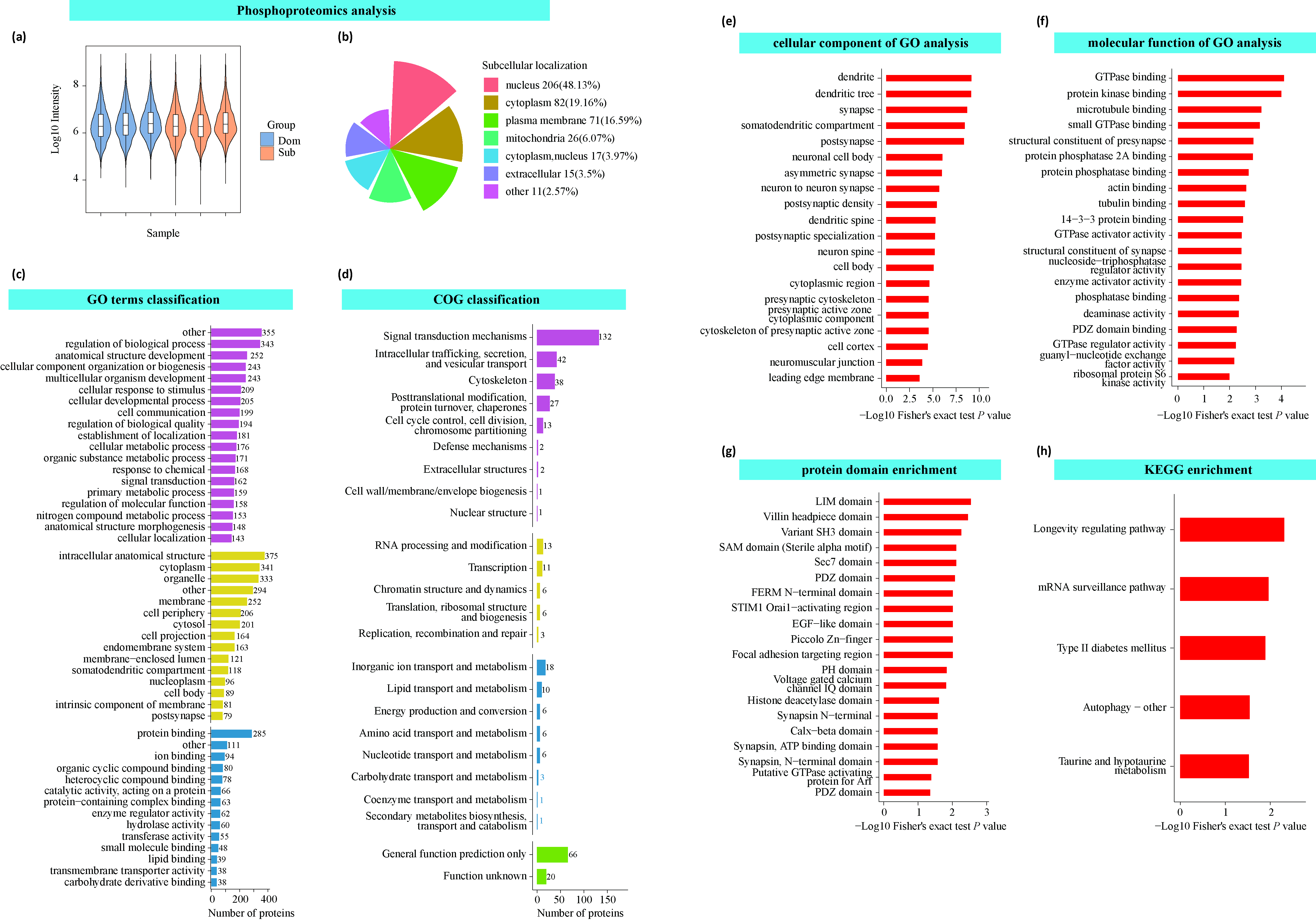

Supplement: Supplementary file 1 [file Image2.tif]

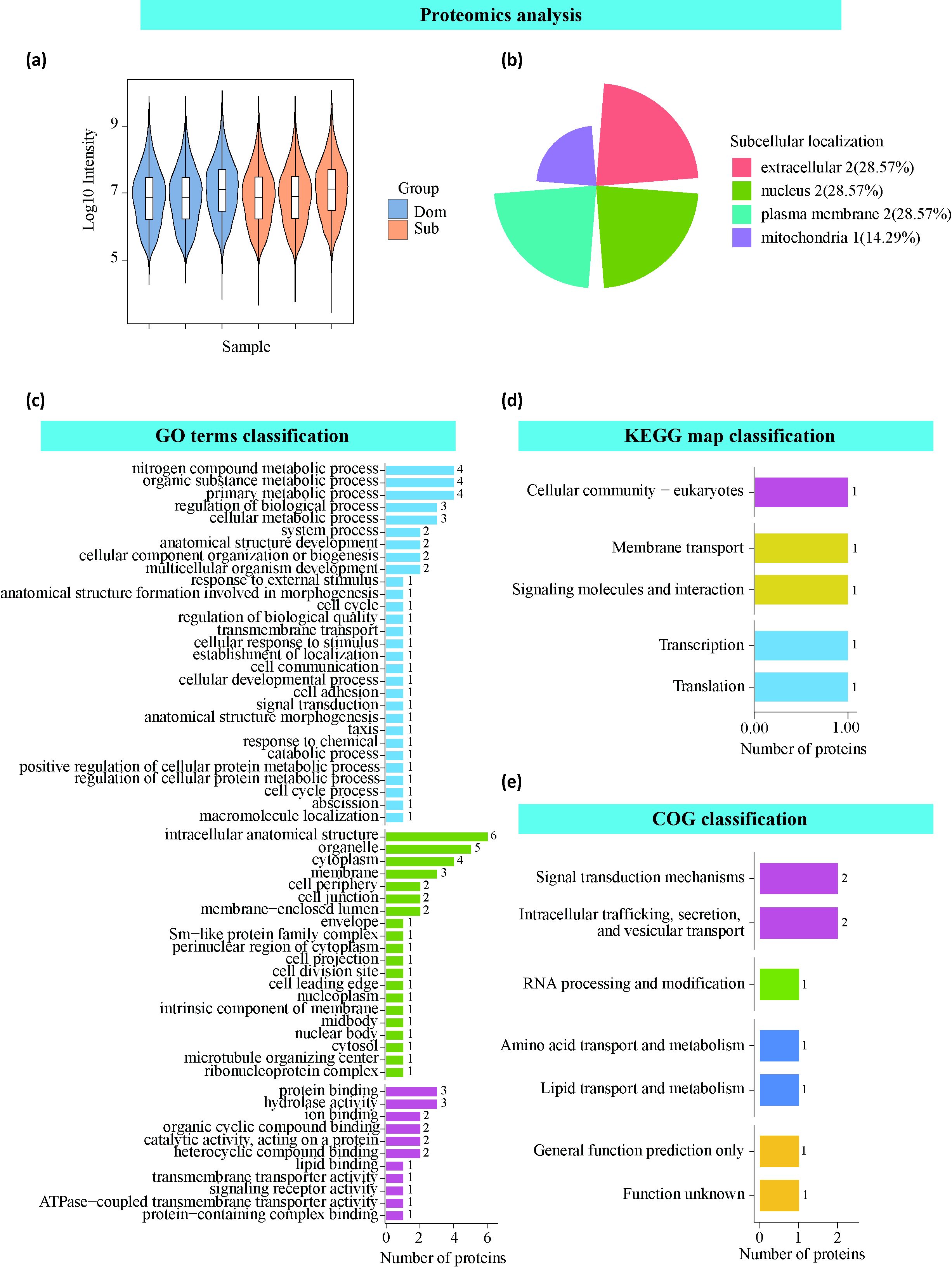

Supplement: Supplementary file 2 [file Image1.tif]
